# Cigarette Brand Preference and Pro-Tobacco Advertising Among Middle and High School Students — United States, 2012–2016

**DOI:** 10.15585/mmwr.mm6704a3

**Published:** 2018-02-02

**Authors:** Siobhan N. Perks, Brian Armour, Israel T. Agaku

**Affiliations:** ^1^Rollins School of Public Health, Emory University, Atlanta, Georgia; ^2^Office on Smoking and Health, National Center for Chronic Disease Prevention and Health Promotion, CDC.

Nearly all adult smokers first try cigarettes before age 18 years (*1*), and adolescents can show symptoms of nicotine dependence within days to weeks of the onset of occasional cigarette smoking (*2*). Having a usual cigarette brand among adolescent smokers could reflect exposure and receptivity to pro-tobacco advertising and tobacco product appeal (*1*). To identify usual cigarette brands smoked among U.S. middle and high school students who were current (past 30-day) cigarette smokers, CDC analyzed data from the 2012–2016 National Youth Tobacco Survey (NYTS). Marlboro, Newport, and Camel were the most commonly reported brands smoked during 2012–2016; in 2016, these three were the brands usually smoked for 73.1% and 78.7% of current cigarette smokers in middle and high school, respectively. These three brands also were the three most commonly identified as having a “favorite cigarette ad” in 2012. Efforts to reduce youth exposure to pro-tobacco advertising could help reduce youth smoking (*1*,*3*).

NYTS is an annual national survey of U.S. students in grades 6–12.* During 2012–2016, sample sizes ranged from 17,711 (response rate = 63.4%) in 2015 to 24,658 (response rate = 73.6%) in 2012 (*4*). Participants were asked, “During the past 30 days, what brand of cigarettes did you usually smoke?” Response options^†^ were “American Spirit,” “Camel,” “GPC, Basic, or Doral,” “Kool,” “Lucky Strike,” “Marlboro,” “Newport,” “Parliament,” “Virginia Slims,” “I did not smoke a usual brand,” “Some other brand not listed here,” “I did not smoke a cigarette in the past 30 days,” and “Not sure.” Responses of “I did not smoke a cigarette in the past 30 days” and “Not sure” were excluded; all other responses were classified as current (past 30-day) cigarette smokers.^§^ Among current cigarette smokers, any response other than “I did not smoke a usual brand” was classified as having a usual brand.

In the 2012 NYTS only, participants were asked, “What is the name of the cigarette brand of your favorite cigarette ad?” Response options were “American Spirit,” “Camel,” “GPC, Basic, or Doral,” “Kool,” “Marlboro,” “Newport,” “Some other brand not listed here,” “I don't have a favorite cigarette ad,” and “Not sure.” Any response other than “I don't have a favorite cigarette ad” and “Not sure” was classified as having a favorite cigarette ad. In the 2015 NYTS only, exposure to ads for both regular cigarettes and electronic cigarettes (e-cigarettes) over four media categories was assessed (the Internet, newspapers/magazines, retail stores, and TV/movies). An exposure was classified as reporting seeing ads on the assessed medium “Sometimes,” “Most of the time,” or “Always.”^¶^ The tobacco product exposed to on each advertising medium was classified as 1) neither e-cigarettes nor cigarettes, 2) e-cigarettes only, 3) cigarettes only, and 4) both e-cigarettes and cigarettes.

Among current cigarette smokers, brand-specific prevalence was calculated overall and by school level, sex, grade, race/ethnicity, and smoking frequency within the past 30 days (a response of 20–30 days was considered frequent; a response of 1–19 days was considered infrequent).** Binary logistic regression was used to assess brand-specific linear trends during 2012–2016, adjusting for grade, sex, and race/ethnicity. For 2012 only, agreement between usual brand and favorite cigarette ad was assessed among 1,807 current cigarette smokers with data available for both indicators. For 2015 only, the proportion of current cigarette smokers reporting having a usual brand^††^ was stratified by amount of reported ad exposure to pro-tobacco advertising across media types. Chi-squared tests and logistic regression were used to determine subgroup differences, with statistical significance set at p<0.05. Data were weighted to yield nationally representative estimates.

During 2016, the top three brands usually smoked among current cigarette smokers in all middle school grades combined were Marlboro (38.3%), Newport (21.4%), and Camel (13.4%) (Table). During 2016, 16.5% of middle school current cigarette smokers smoked some other specific brand, and 10.4% had no usual brand. The proportion of current cigarette smokers who smoked Marlboro cigarettes during 2016 was highest among non-Hispanic whites (whites) (54.6%) and lowest among non-Hispanic blacks (blacks) (11.5%; p<0.05). Conversely, the proportion who smoked Newport cigarettes during 2016 was highest among blacks (58.4%) and lowest among whites (7.9%; p<0.05). A higher proportion of female smokers (27.2%) smoked Newport cigarettes than did male smokers (16.6%; p<0.05). Trends during 2012–2016 were not significant for middle school students overall or among subgroups.

**TABLE:** Brand of cigarettes usually smoked by current (past 30-day)* cigarette smokers in middle and high school, by selected characteristics — National Youth Tobacco Survey, United States, 2012–2016^†^

Characteristic	Marlboro		Newport		Camel		Other specific brand^§^		No usual brand	
	2012	2016	2012	2016	2012	2016	2012	2016	2012	2016
	% (SE)	% (SE)	% (SE)	% (SE)	% (SE)	% (SE)	% (SE)	% (SE)	% (SE)	% (SE)
**Middle school**										
Total	37.0 (3.5)	38.3 (4.1)	17.1 (2.4)	21.4 (3.5)	17.8 (2.8)	13.4 (2.4)	17.5 (2.2)	16.5 (2.4)	10.5 (1.6)	10.4 (1.8)
**Sex**										
Male	38.0 (4.5)	38.9 (6.0)	14.6 (2.7)	16.6 (3.8)	19.7 (3.8)	14.5 (3.5)	18.0 (2.7)	17.3 (3.9)	9.7 (1.9)	12.6 (2.7)
Female	35.7 (3.9)	37.2 (4.6)	20.5 (3.2)	27.2 (4.3)	15.4 (2.7)	12.3 (2.9)	16.9 (3.0)	15.6 (3.6)	11.6 (2.1)	7.6 (2.4)
**Grade**										
6	33.8 (4.9)	40.6 (6.3)	19.7 (4.0)	17.4 (4.6)	15.8 (2.8)	13.4 (4.4)	20.7 (4.5)	18.7 (4.6)	10.1 (2.8)	9.9 (3.6)
7	38.4 (5.9)	33.2 (4.8)	16.3 (3.6)	22.5 (4.6)	16.7 (4.1)	15.8 (3.4)	17.8 (3.7)	13.4 (3.3)	10.8 (2.2)	15.1 (3.5)
8	37.6 (3.8)	41.4 (6.2)	16.5 (2.3)	22.4 (4.7)	19.6 (3.8)	11.5 (3.0)	15.8 (3.2)	17.9 (3.6)	10.6 (2.2)	6.9 (1.9)
**Race/Ethnicity**										
White, non-Hispanic	44.3 (4.8)	54.6 (5.1)	8.5 (2.1)	7.9 (2.8)	20.3 (5.0)	16.1 (3.5)	17.5 (3.3)	9.4 (3.2)	9.4 (2.3)	12.1 (3.6)
Black, non-Hispanic	28.4 (6.9)	11.5 (5.1)	42.7 (6.6)	58.4 (5.6)	3.8 (0.9)	8.6 (4.3)	16.7 (4.8)	15.5 (5.4)	8.4 (3.7)	6.0 (2.8)
Hispanic	33.2 (4.2)	26.5 (4.2)	14.9 (2.6)	21.3 (5.9)	20.8 (5.5)	18.5 (4.4)	18.8 (4.6)	23.8 (5.2)	12.4 (3.0)	9.9 (3.2)
**No. of days smoked in past 30 days** ^¶^										
Frequent (≥20 days)	44.8 (9.2)	47.5 (11.0)	14.8 (4.0)	9.1 (4.8)	17.8 (6.5)	14.7 (7.9)	19.5 (6.8)	26.6 (9.4)	3.0 (2.2)	2.0 (2.0)
Infrequent (1–19 days)	41.6 (4.8)	40.3 (7.6)	19.0 (3.7)	18.6 (5.3)	16.1 (4.1)	17.3 (4.0)	18.5 (3.1)	14.0 (4.5)	4.8 (1.2)	9.9 (4.1)
**High school**										
Total	38.5 (1.8)	48.8 (2.4)**	23.1 (2.1)	16.6 (1.8)**	17.8 (1.4)	13.3 (1.3)**	16.4 (1.5)	15.4 (1.6)	4.1 (0.4)	5.9 (0.9)**
**Sex**										
Male	39.4 (2.1)	50.0 (2.8)**	21.0 (2.0)	16.0 (2.2)	17.0 (1.5)	12.5 (1.7)**	17.4 (1.8)	15.6 (2.1)	5.1 (0.7)	5.8 (1.2)
Female	37.5 (2.3)	48.0 (3.5)**	26.0 (2.7)	16.8 (2.4)**	18.6 (2.1)	14.2 (1.9)**	15.2 (1.7)	15.0 (1.9)	2.7 (0.5)	6.0 (1.2)**
**Grade**										
9	34.3 (2.6)	42.9 (3.7)**	25.1 (2.7)	18.4 (2.8)	17.4 (2.2)	15.9 (3.6)	16.2 (1.5)	17.4 (3.1)	6.9 (1.4)	5.4 (1.5)
10	37.2 (2.4)	45.7 (3.7)**	25.5 (3.1)	19.5 (3.0)	19.4 (2.3)	14.2 (3.9)**	14.9 (1.8)	13.9 (1.7)	2.9 (0.7)	6.8 (2.3)**
11	40.3 (2.7)	50.8 (4.4)	22.5 (2.7)	17.2 (3.1)	14.5 (1.6)	10.0 (1.9)	19.0 (2.2)	15.6 (1.5)	3.8 (0.8)	6.4 (1.5)
12	41.1 (2.5)	53.2 (3.7)**	20.3 (2.4)	12.7 (2.0)	19.8 (2.5)	13.6 (1.8)**	15.5 (2.9)	15.3 (2.6)	3.3 (0.6)	5.1 (1.2)**
**Race/Ethnicity**										
White, non-Hispanic	45.8 (2.1)	59.5 (3.1)**	15.4 (1.8)	9.5 (1.6)**	19.6 (1.9)	11.9 (1.9)**	15.4 (2.0)	14.1 (2.1)	3.7 (0.6)	5.0 (1.4)
Black, non-Hispanic	10.3 (2.7)	11.0 (3.6)	67.0 (4.3)	47.5 (7.6)	4.2 (1.7)	8.9 (3.0)	16.9 (2.7)	16.7 (5.6)	1.6 (0.7)	15.9 (2.5)**
Hispanic	36.6 (2.6)	40.5 (3.2)	20.5 (3.0)	20.2 (3.3)	20.7 (2.3)	18.1 (2.1)	17.8 (2.3)	16.5 (2.0)	4.4 (1.3)	4.7 (1.4)
**No. of days smoked in past 30 days** ^¶^										
Frequent (≥20 days)	42.2 (2.8)	59.1 (5.1)**	25.6 (2.9)	12.5 (3.4)	18.2 (2.3)	14.0 (2.7)	12.7 (1.9)	11.5 (2.7)	1.3 (0.4)	2.9 (1.3)
Infrequent (1–19 days)	37.8 (2.4)	50.8 (3.5)**	21.6 (2.3)	17.1 (2.5)	19.8 (2.3)	12.4 (2.2)**	18.0 (2.2)	16.6 (2.2)	2.8 (0.6)	3.1 (1.1)

**Abbreviation**: SE = standard error.

* Assessed with the question: “During the past 30 days, what brand of cigarettes did you usually smoke?” Response options were “American Spirit,” “Camel,” “GPC, Basic, or Doral,” “Kool,” “Lucky Strike,” “Marlboro,” “Newport,” “Parliament,” “Virginia Slims,” “I did not smoke a usual brand,” “Some other brand not listed here,” “I did not smoke a cigarette in the past 30 days,” and “Not sure.” Any response other than “I did not smoke a cigarette in the past 30 days” or “Not sure” was treated as being a current (past 30-day) cigarette smoker.

^†^ Trend analyses include data for 2012, 2013, 2014, 2015, and 2016. Prevalence estimates are presented only for 2012 and 2016.

^§^ Because of small sample sizes, the responses “GPC, Basic, or Doral,” “Kool,” “Lucky Strike,” “Parliament,” and “Virginia Slims” were combined together as one category (“Other specific brand”).

^¶^ Assessed with the question “During the past 30 days, on how many days did you smoke cigarettes?” Response options included “0 days,” “1 or 2 days,” “3 to 5 days,” “6 to 9 days,” “10 to 19 days,” “20 to 29 days,” and “All 30 days.” Responses of “0 days” were excluded. All other responses were dichotomized as frequent (≥20 days) or infrequent (1–19 days).

** Statistically significant linear trend during 2012–2016 (p-trend<0.05).

Among high school current cigarette smokers, the top three brands usually smoked by students in all grades combined in 2016 also were Marlboro (48.8%), Newport (16.6%), and Camel (13.3%) (Table). During 2016, 15.4% of high school current cigarette smokers smoked other specific brands, and 5.9% reported no usual brand. As was the case among middle school students, Newport was the most prevalent brand among black high school students (47.5% in 2016), and Marlboro was the most prevalent brand among white high school students (59.5% in 2016). During 2016, the proportion of high school current cigarette smokers that smoked Camel cigarettes was highest among Hispanics (18.1%) and lowest among blacks (8.9%). Trend analyses during 2012–2016 indicated an increase in the prevalence of Marlboro smoking for all high school students (38.5% to 48.8%), males (39.4% to 50.0%), females (37.5% to 48.0%), ninth graders (34.3% to 42.9%), 10th graders (37.2% to 45.7%), 12th graders (41.1% to 53.2%), whites (45.8% to 59.5%), and both frequent (42.2% to 59.1%) and infrequent smokers (37.8% to 50.8%) (all p-values for trend <0.05). The prevalence of Newport smoking declined during 2012–2016 among all high school students (23.1% to 16.6%), females (26.0% to 16.8%), and whites (15.4% to 9.5%) (all p-values for trend <0.05). The prevalence of Camel smoking during 2012–2016 declined among all high school students (17.8% to 13.3%), males (17.0% to 12.5%), females (18.6% to 14.2%), 10th graders (19.4% to 14.2%), 12th graders (19.8% to 13.6%), whites (19.6% to 11.9%), and infrequent smokers (19.8% to 12.4%) (all p-values for trend <0.05). The proportion of students who smoked no usual brand increased among all high school students (4.1% to 5.9%), females (2.7% to 6.0%), 10th graders (2.9% to 6.8%), 12th graders (3.3% to 5.1%), and blacks (1.6% to 15.9%) during 2012–2016 (all p-values for trend <0.05).

In 2012, among current cigarette smokers who reported smoking a usual brand, 72.1% identified the same brand as their favorite cigarette ad. The top three favorite cigarette ads were also the top three brands usually smoked (Figure 1).

**FIGURE 1:**
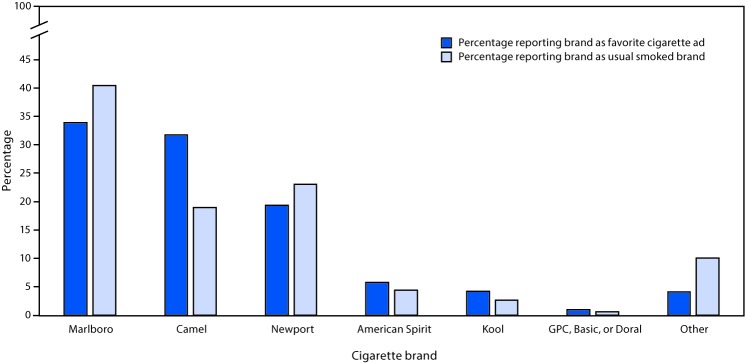
Agreement* between brand of cigarettes usually smoked^†^ and favorite cigarette brand ad^§^ among middle and high school current (past 30-day) cigarette smokers — National Youth Tobacco Survey, United States, 2012 * Restricted to students who smoked cigarettes during the past 30 days and reported having both a favorite cigarette ad and a cigarette brand usually smoked (n = 1,807). The question on favorite cigarette ad was asked only in 2012. ^†^ Assessed with the question: “During the past 30 days, what brand of cigarettes did you usually smoke?” Responses classified as having a brand usually smoked among past 30-day smokers included “American Spirit,” “Camel,” “GPC, Basic, or Doral,” “Kool,” “Lucky Strike,” “Marlboro,” “Newport,” “Parliament,” “Virginia Slims,” and “Some other brand not listed here.” ^§^ Assessed with the question: “What is the name of the cigarette brand of your favorite cigarette ad?” Responses classified as having a favorite cigarette ad were “American Spirit,” “Camel,” “GPC, Basic, or Doral,” “Kool,” “Marlboro,” “Newport,” and “Some other brand not listed here.”

In 2015, across all advertising media, current cigarette smokers who reported exposure to neither e-cigarette ads nor cigarette ads reported significantly lower prevalence of having a usual brand than those who reported exposure to both ads (Figure 2). By specific advertising media, among those exposed to neither e-cigarette nor cigarette ads versus both ads, the proportion who reported having a usual brand was as follows: for movies/TV (neither ad = 80.5%; both ads = 94.2%), for retail stores (neither = 69.8%; both = 94.8%), for Internet (neither = 79.4%; both = 94.5%), and for magazines/newspapers (neither = 88.0%; both = 94.6%) (all p-values <0.05).

**FIGURE 2:**
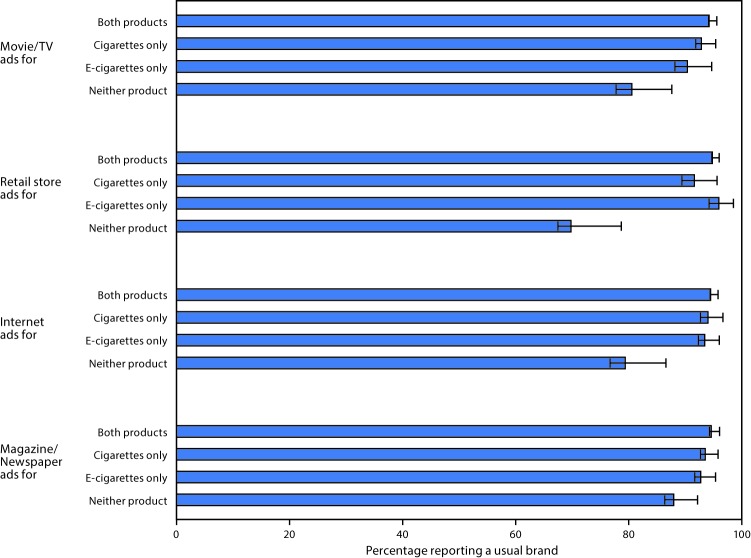
Proportion of middle and high school current (past 30-day) cigarette smokers reporting a usual cigarette brand,* by advertising medium and status of exposure to cigarette and/or electronic cigarette ads^†^ — National Youth Tobacco Survey, United States, 2015^§^ * Outcome was dichotomized as 0 or 1. Persons who reported having a specific brand they usually smoked (“American Spirit,” “Camel,” “GPC, Basic, or Doral,” “Kool,” “Lucky Strike,” “Marlboro,” “Newport,” “Parliament,” “Virginia Slims,” or “Some other brand not listed here”) were coded as 1. Those who responded, “I did not smoke a usual brand” were coded as 0. Responses of “Not sure” or “I did not smoke a cigarette in the past 30 days” were excluded. ^†^ Separate questions were asked for electronic cigarettes and regular cigarettes in relation to exposure to pro-tobacco ads on the different media sources (Internet, newspapers/magazines, retail stores, and TV/movies). For both electronic cigarettes and regular cigarettes, respondents’ ad exposure status was coded on each medium as either: 1 = exposed (responses of “Sometimes,” “Most of the time,” and “Always”) or 0 = nonexposed (“Never,” “Rarely,” or those who indicated not using the assessed medium). ^§^ The questions on exposure to both electronic cigarette and regular cigarette ads were asked only in 2015.

## Discussion

During 2012–2016, the top three brands usually smoked by U.S. middle and high school current cigarette smokers were Marlboro, Newport, and Camel; these brands also were the top three favorite cigarette ads reported by current cigarette smokers in middle and high school in 2012. Market data also indicated that these three brands accounted for the largest share (62%) of the U.S. cigarette market during 2016; the percentage shares of retail volume for Marlboro, Newport, and Camel during 2016 were 40.2%, 13.8%, and 8.0% respectively (*5*). Cigarette ads use youth-oriented themes, including those highlighting independence, rebellion, and perceived social acceptability of cigarette smoking (*3*). Previous epidemiologic studies have demonstrated an association between amount of reported ad exposure and most frequently smoked brands among adolescents (*6*); efforts to reduce youth exposure to pro-tobacco advertising might help reduce smoking initiation among U.S. youth (*1*).

Targeted marketing of tobacco products to certain groups can explain differences in brand preferences among subgroups (*1*,*7*,*8*). Whereas Marlboro smoking was more prevalent among whites, Newport, a predominantly menthol brand, was more often smoked by blacks, which is consistent with previous reports that have documented that menthol cigarettes are marketed to specific demographic groups, including blacks (*7*,*8*). Among high school students overall, as well as among females, blacks, and 10th and 12th graders, significant increases were observed in the proportion of smokers reporting no usual brand. Having no usual brand might be an indicator of nonspecific cigarette access patterns, including from social sources such as friends (*1*).

The findings in this report are subject to at least four limitations. First, self-reported cigarette smoking is subject to social desirability bias and might be underreported among youth. Second, both brand preferences and pro-tobacco ad exposure were measured at the same time in this cross-sectional study; the data therefore did not permit assessment of temporality. Exposure to ads could increase brand use or brand use could lead to a favorable impression of tobacco ads. Third, these findings might not be generalizable to youth who are not enrolled in traditional schools, (e.g., dropouts [approximately 6.4% among high school students]^§§^ and those home-schooled [approximately 3.4% of school-aged children]).^¶¶^ Finally, the relationships between “favorite cigarette ad” and cigarette brand preferences as assessed in 2012 NYTS might have limited comparability with subsequent years.

In 2014, U.S. cigarette manufacturers spent approximately $8.5 billion, or approximately $1 million per hour, to advertise and promote cigarettes (*9*). Information on cigarette brand usually smoked can help guide efforts to reduce cigarette smoking among the approximately 1.6 million U.S. middle and high school cigarette smokers (*10*). Reducing youth-oriented tobacco marketing, as part of a comprehensive approach in concert with other evidence-based strategies could help reduce the acceptability, affordability, and use of tobacco products among youth (*1*). Such strategies include comprehensive smoke-free policies, increasing the prices of tobacco products, and raising the minimum age of purchase for tobacco products to 21 years (*1*).

**Summary**

**What is already known about this topic?**

Nearly all adult smokers first try cigarettes before age 18 years. Tobacco-advertising activities, among other factors, including peer influence and price, are associated with initiation of smoking and the continued use of tobacco products among youth.

**What is added by this report?**

Analysis of 2012–2016 National Youth Tobacco Survey data found that Marlboro, Newport, and Camel were the most commonly reported usual brands smoked by middle and high school current (past 30-day) cigarette smokers. In 2016, these three brands accounted for 73.1% and 78.7% of current cigarette smokers in middle and high school, respectively. Ads for these three brands were also the three most commonly identified “favorite cigarette ad” in 2012. Current cigarette smokers who reported exposure to neither e-cigarette ads nor cigarette ads reported significantly lower prevalence of having a usual brand than those who reported exposure to both ads during 2015.

**What are the implications for public health practice?**

Reducing youth-oriented tobacco marketing, as part of a comprehensive approach in concert with other evidence-based strategies, including comprehensive smoke-free policies, increasing the price of tobacco products, and raising the minimum age of purchase for tobacco products to 21 years, could help reduce the acceptability, affordability, and use of tobacco products among youth.

## Footnotes

* The study period was restricted to 2012–2016 because the questions assessing cigarette brand usually smoked had different response options in preceding NYTS survey years.

† Because of small sample sizes, “GPC, Basic, or Doral,” “Kool,” “Lucky Strike,” “Parliament,” and “Virginia Slims” were collapsed together into one category (“Other specific brand”).

§ Final analytical sample for each year (past 30-day cigarette smokers) was as follows: 2012 (n = 3,292), 2013 (n = 2,377), 2014 (n = 2,386), 2015 (n = 1,823), and 2016 (n = 1,739).

¶ For each specific advertising medium assessed, participants could select any one of the following response options that best described their frequency of exposure: “Never,” “Rarely,” “Sometimes,” “Most of the time,” or “Always.” Participants could also indicate if they did not use the medium assessed (e.g., “I do not use the Internet”). Participants who answered “Never” or “Rarely,” or who indicated they did not use the assessed medium, were classified as nonexposed to that medium; all other responses were classified as exposed.

** Frequency of cigarette smoking was ascertained with the question “During the past 30 days, on how many days did you smoke cigarettes?” Categorical response options were **“**0 days,” “1 or 2 days,” “3 to 5 days,” “6 to 9 days,” “10 to 19 days,” “20 to 29 days,” and “All 30 days.” A response of “0 days” was classified as being a current nonsmoker and was excluded. The remaining response options were dichotomized as infrequent (1–19 days) and frequent (≥20 days) cigarette smokers.

†† Outcome was dichotomized as 0 or 1. Persons who reported having a specific brand they usually smoked (“American Spirit,” “Camel,” “GPC, Basic, or Doral,” “Kool,” “Lucky Strike,” “Marlboro,” “Newport,” “Parliament,” “Virginia Slims,” or “Some other brand not listed here”) were treated as a positive response. Those who responded, “I did not smoke a usual brand” were treated as not having a brand usually smoked. Responses of “Not sure” or “I did not smoke a cigarette in the past 30 days” were excluded.

§§
https://www.census.gov/newsroom/press-releases/2016/cb16-tps142.html .

¶¶ https://nces.ed.gov/programs/digest/d15/tables/dt15_206.10.asp?current=yes.
